# Low-Dose Abdominal CT for Evaluating Suspected Appendicitis: Recommendations for CT Imaging Techniques and Practical Issues

**DOI:** 10.3390/diagnostics12071585

**Published:** 2022-06-29

**Authors:** Ji Hoon Park, Hae Young Kim, Ji Ye Sim, Kyoung Ho Lee

**Affiliations:** 1 Department of Radiology, Seoul National University Bundang Hospital, Seongnam-si 13620, Korea; pjihoon79@gmail.com (J.H.P.); qkfmrp860329@gmail.com (H.Y.K.); 2 Department of Applied Bioengineering, Graduate School of Convergence Science and Technology, Seoul National University, Seoul 08826, Korea; 3 Department of Radiology, Seoul National University College of Medicine, Seoul National University Bundang Hospital, Seongnam-si 13620, Korea; 4 Department of Radiology, Hanil General Hospital, Seoul 01450, Korea; sunnyandluna@naver.com; 5 Interdisciplinary Program in Bioengineering, Seoul National University, Seoul 08826, Korea

**Keywords:** abdomen, acute, appendicitis, multidetector computed tomography, patient care team, quality control, radiation, ionizing

## Abstract

A vast disparity exists between science and practice for CT radiation dose. Despite high-level evidence supporting the use of low-dose CT (LDCT) in diagnosing appendicitis, a recent survey showed that many care providers were still concerned that the low image quality of LDCT may lead to incorrect diagnoses. For successful implementation of LDCT practice, it is important to inform and educate the care providers not only of the scientific discoveries but also of concrete guidelines on how to overcome more practical matters. Here, we discuss CT imaging techniques and other practical issues for implementing LDCT practice.

## 1. Introduction

CT radiation used for the diagnosis of acute appendicitis is potentially carcinogenic [1,2]. Despite high-level evidence, including that from two large randomized controlled trials [3,4], a vast disparity exists between science and practice for CT radiation dose. A recent survey showed that many of the care providers were still concerned that the low image quality of LDCT may lead to inaccuracies in diagnosing appendicitis [5].

For successful implementation of LDCT in clinical practice, an understanding of scientific evidence supporting LDCT is essential, but real challenges sometimes lie in the practical issues. A review article [2] is available for evidence supporting the need of reducing CT radiation. Here, we limit our review to the technical aspects specifically of low-dose appendiceal CT and other practical issues for implementing LDCT practice.

## 2. Technical Consideration

### 2.1. Intravenous Contrast Enhancement

We recommend using intravenous contrast enhancement, which is essential to compensate for the low image quality of LDCT. Although debatable, the ACR Appropriateness Criteria [6] now recommends using intravenous contrast enhancement for the diagnosis of appendicitis. However, this guideline was based in earlier studies that used conventional-dose CT (CDCT), and there have been few investigations on the need for intravenous contrast enhancement in LDCT. Several studies [7,8,9,10] have reported that precontrast LDCT (1–4 mSv) was comparable to contrast-enhanced CDCT (5–10 mSv) in the diagnosis of appendicitis. However, these studies also found that unenhanced LDCT may be limited for alternative diagnoses [8,10], diagnostic confidence for appendicitis [10], or visualization of the normal appendix [10].

### 2.2. Contrast-Enhancement Phase

We recommend obtaining portal venous phase images only. We are not aware of any published evidence suggesting that any additional precontrast, arterial phase, or delayed phase images are helpful for patients with suspected appendicitis. Guidelines including the ACR Appropriateness Criteria [6] have not specifically addressed this issue. A Korean survey in 2011 [11] surprisingly showed that 10 of the 11 teaching hospitals were routinely acquiring either precontrast or arterial phase images or both in addition to portal venous phase images in appendiceal CT.

Radiologists and referring physicians or surgeons are often reluctant to abandon precontrast CT or multiphase scanning. The reluctance is often due to the concern of a missed diagnosis of a urinary stone, which is an important alternative diagnosis [12]. A recent study [13] reported that three radiologists’ retrospective reading of portal-venous-phase images showed sensitivities ranging 92%–96% in detecting urinary stones larger than 3 mm. Smaller stones were more prone to be missed, although they generally do not require any invasive treatment procedures [14].

### 2.3. Enteric Contrast

We recommend against using enteric contrast in LDCT. As the ACR Appropriateness Criteria [6] states, evidence is trending against the use of enteric contrast for intravenous contrast-enhanced CT. A large observational study [15] from the United States showed that the use of enteric contrast does not improve the diagnosis of appendicitis. These guidelines and study results are based on data obtained using CDCT, and there have been few investigations on the need for enteric contrast in LDCT.

### 2.4. Anatomical Coverage

Several researchers [16,17,18] have proposed limiting scan coverage to the pelvis in appendiceal CT. However, this “focused” CT was criticized by other researchers [19,20] who showed that it could lead to some missed diagnosis of appendicitis or other critical abnormalities outside the pelvis. The ACR Appropriateness Criteria [6] recommend scanning the upper abdomen as well as the pelvis in patients with suspected appendicitis.

In practice, scanning beyond the intended range (i.e., “image creep”) often occurs in abdomen CT [21], which leads to higher doses. In most CT machines, the scan range cannot be set as a part of an automated scan program but needs to be adjusted manually by technologists. Therefore, we recommend specifying anatomical landmarks for determining the scan coverage from scout images and using those landmarks consistently. For example, we set the scan range from 4 cm above the liver dome to 1 cm below the ischial tuberosity.

### 2.5. Tube Current

Reducing tube current has been the mainstay of dose-reducing techniques in previous LDCT studies. As stated above, those studies have proved excellent diagnostic and clinical outcomes with LDCT using reduced tube currents. In our centers, we set the reference value for an effective tube-current-time product as 45–110 mAs, aiming at effective radiation doses of 2 mSv. This wide range is primarily due to variation in the tube potentials used in individual CT machines and hospitals. We recommend activating all available automatic exposure control techniques in the automated scan program that is saved in each CT machine. In spite of the advantages of automatic exposure control techniques [22], the 2011 survey by Park and colleagues [11] showed that one of the 11 teaching hospitals inadvertently failed to use the automatic exposure control technique in some patients.

### 2.6. Tube Potential

The tube potential for standard abdomen CT for adults has been typically 120 kVp or 140 kVp. It is now widely accepted that lower tube potentials (80–100 kVp) can be used for smaller adults [23]. While it is desirable to individualize tube potential by patient size automatically [24] or manually, the 2011 Korean survey [11] showed that all 11 participating sites were using fixed tube potentials: 120 kVp at ten sites and 100 kVp at one site.

### 2.7. Iterative Reconstruction

**Figure 1:**
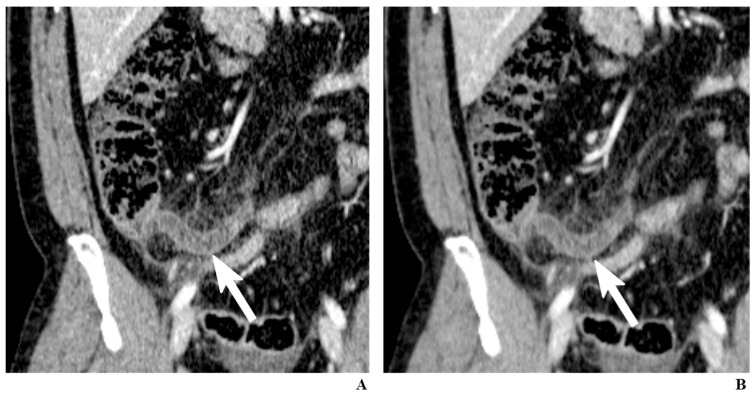
A 34-year-old man with appendicitis. Contrast-enhanced coronal CT images reconstructed by filtered back-projection (FBP) (**A**) and iterative reconstruction (IR) (**B**). While both images clearly depict an inflamed appendix (arrows), the image reconstructed using IR generates less noise, which can alleviate the practitioners’ reluctance to low-dose CT.

Many studies have advocated that using an iterative reconstruction instead of a filtered back-projection can allow considerable dose reduction without significant sacrifice in image quality [25]. However, few studies have investigated whether an iterative reconstruction is truly helpful in low-dose appendiceal CT. Park and colleagues [26] retrospectively compared a filtered back-projection and an iterative reconstruction in 107 patients who underwent 2 mSv CT for suspected appendicitis. Interestingly, the researchers did not find any notable advantage of the iterative reconstruction over the filtered back-projection in the diagnostic performance or diagnostic confidence, although radiologists assigned higher subjective image-quality scores for the iterative reconstruction than for the filtered back-projection (Figure 1). In a more recent prospective study [27], the same researcher group showed that the radiation dose of appendiceal CT could be lowered to 0.5 mSv by using a new-generation iterative reconstruction technique.

### 2.8. Image Reconstruction Thickness

As the appendix is a small structure, it has been believed that thinner sections are advantageous in depicting the normal or inflamed appendix. A decade ago, Johnson and colleagues [28] reported that appendiceal visualization improved with decreased section thickness from 5 mm to 2 mm at CDCT (using an effective tube-current-time product of 200 mAs and a tube potential of 120 kVp). With LDCT using a dose of around 2 mSv, however, the conventional wisdom that thinner sections are advantageous may not be valid. As image noise is inversely correlated with the number of X-ray photons that contribute to the formation of that image [29], decreasing section thickness increases image noise further. Considering the trade-off between *z*-axis spatial resolution and image noise, we recommend 3–5 mm as the viewing thickness (i.e., section thickness) for LDCT with a dose of around 2 mSv, based on our experience from the two large trials [3,4]. In our centers, we reconstruct two transverse image datasets from each helical scan: 4 mm thickness with 3 mm interval and 2 mm thickness with 1 mm interval. We primarily review the 4 mm thick images and occasionally use the 2 mm thick images for multiplanar sliding-slab averaging review, which we will discuss later.

### 2.9. Coronal Reformation

In a retrospective study using CDCT (tube-current-time product of 350 mA and tube potential of 140 kVp), Paulson and colleagues [30] reported that coronal reformations used in addition to transverse images enhanced radiologists’ diagnostic confidence, which was measured with a scale of 1 to 5 confidence score (1, definitely absent; 2, probably absent; 3, cannot determine; 4, probably present; 5, definitely present), but did not improve diagnostic performance for appendicitis significantly. We are not aware of any study that has formally measured the advantage of additional coronal reformations in LDCT. The advantages of additional coronal reformations may be theoretically more pronounced for LDCT, given that better appendiceal visualization by additional coronal reformations may compensate for the low image quality of LDCT.

### 2.10. Sliding-Slab Averaging Technique

Sliding-slab averaging technique is a real-time image rendering technique that is useful for rapidly reviewing large thin-section datasets. While the viewing slab slides through the volume along a viewing direction in a small increment, the overlapping slabs create an illusion of image-to-image continuity, thereby preserving the high through-plane spatial resolution that is inherent to a thin-section dataset (Videos S1 and S2 in Supplementary Materials). With the flexibility that allows a reviewer to arbitrarily choose the slab thickness and viewing direction, the dynamic navigation technique is theoretically more advantageous compared to adding simple coronal reformations, particularly in tracing small tortuous tubular structures such as the appendix. Lee and colleagues [31] introduced sliding-slab averaging technique in appendiceal CT. In their retrospective study using CDCT (unspecified tube current and tube potential of 120 kVp), the sliding-slab averaging review of 2 mm thick sections outperformed the regular stack review of 5 mm thick transverse sections in radiologists’ diagnostic confidence, although the difference in diagnostic performance did not reach a statistical significance.

Similar results were found with LDCT in a subsequent retrospective study [32] by the same researcher group. In theory, the sliding-slab averaging technique may be particularly helpful for LDCT because averaging voxels within the slab improves the quality of the rendered images by canceling out the image noise of the thin-section source images. As mentioned above, we recommend keeping slab thickness as 3–5 mm in reviewing LDCT, since a very thin slab would have too much image noise.

## 3. Other Practical Issues in Implementing LDCT

As we mentioned earlier, a vast disparity exists between science and practice for CT radiation dose [33]. Acknowledging this challenge, we designed the multi-center randomized clinical trial [4] as a pragmatic trial, with the intention that the participating sites would eventually embed 2 mSv CT into their usual care by implementing the trial protocol [34]. First, the eligibility criteria (i.e., patients aged 15–44 years undergoing CT due to suspected appendicitis) were broad and largely dependent on the judgment of individual care providers. Second, we minimized the requirements for the CT imaging and interpretation protocol. Third, all co-interventions (i.e., diagnostic and therapeutic procedures other than the initial appendiceal CT) followed the standard practice of each site without using extra resources.

Despite extensive efforts over the years of the trial design and conduct, follow-up results regarding LDCT adoption in the trial sites were not very satisfactory according to a survey [5] conducted during the final phase of the trial. The survey of 579 care providers from the 20 trial sites showed that 7.9% of the care providers were still unwilling to use 2 mSv CT, while the remaining care providers supported consistent (27.3%) or selective (e.g., during working hours) (64.8%) use of 2 mSv CT. The survey showed that many of the care providers were still concerned that the low image quality of LDCT may lead to incorrect diagnoses. It is disappointing that those care providers were still unaware of or disregarded previous study results showing that LDCT is comparable to CDCT in both diagnostic performance [35,36,37] and clinical outcomes [3]. A follow-up survey in 2017 [5] conducted six months after the trial completion showed that six of the 20 participating sites were using the standard-of-care radiation doses of 4 mSv or higher, while the remaining 14 hospitals lowered the dose to 2 mSv. These survey results are partly disappointing given that all 20 sites are highly-resourced teaching hospitals that voluntarily participated in the trial [3]. Our experience shows difficulties in implementing LDCT practice in reality. In addition to an understanding of the theories and imaging techniques of LDCT, real challenges lie in the practical issues that we discuss below.

### 3.1. Dedicated Protocol for Appendiceal CT

For successful implementation of LDCT practice, we strongly recommend first setting up a dedicated appendiceal CT protocol in the hospital information system and the corresponding automated scan program in each CT machine. This automation and standardization are particularly crucial in hospitals wherein routine workflow does not allow radiologists to determine the scanning protocol for each individual patient, or in large hospitals where not all care providers are enthusiastic about the dose reduction. Setting up a dedicated CT protocol can be a starting point to identify the components that should be reinforced or revised in the CT examination cycle, spanning from the order entry to the report of the results. For example, a simple query to the hospital information system can identify care providers who are reluctant towards the shift from the general-purpose CDCT to the dedicated appendiceal LDCT. Those reluctant care providers could be the primary target of further education and encouragement, as we discuss later.

The 2017 survey [5] conducted six months after the completion of the multi-center randomized controlled trial [4] showed that only four of the 20 trial sites were consistently using the dedicated appendiceal CT protocol for adolescents and young adults with suspected appendicitis. Six sites were selectively using the dedicated protocol, and ten abandoned the dedicated protocol from their usual practice. Although partly disappointing, these results were still a remarkable progression from the 2011 survey by Park and colleagues [11], which showed that only one of the 11 participating hospitals had a dedicated appendiceal CT protocol.

### 3.2. Education for Referring Physicians and Surgeons

It is understandable that some referring physicians, surgeons, and even radiologists are not enthusiastic or are even reluctant towards dose reduction. Care providers’ actions are often unfortunately influenced by the concern of malpractice litigation. In the United States, appendicitis is one of the most common medical conditions associated with litigation against emergency department physicians, with up to one-third of cases ending up with claims paid to patients [38,39]. The risk of an inaccurate diagnosis of appendicitis due to degraded image quality by using inadequate radiation dose can immediately affect care providers as well as patients. On the contrary, the potential risk of carcinogenesis due to excessive radiation is so small and unlikely to be immediate that the risk may rarely affect the care providers’ choice of the CT examination. Therefore, it is essential to create higher-level evidence supporting the dose reduction and to educate colleague physicians, surgeons, and radiologists on such evidence. The education can occur through lectures, printed material, institutional and societal websites, individual consultations by radiologists or physicists to referring clinicians, and the use of decision support in order entry [40].

### 3.3. Education for Radiologists

With higher image-noise level, LDCT images are often less straightforward to interpret than CDCT images are, especially for inexperienced radiologists. Two studies [3,41] reported that radiologists’ diagnostic confidence tended to be lower with LDCT than with CDCT, although the observed difference did not reach statistical significance. In a prospective study by Yang and colleagues [42], 63 attending radiologists and 166 radiologist residents from 22 hospitals with little prior experience in using LDCT completed an online training course consisting of 30 cases of 2 mSv CT images with direct feedback. Interestingly, these data did not show notable intrareader learning curves over the 30 cases. Instead, diagnostic performance was affected rather by readers’ years of overall clinical experience and prior experience with appendiceal CT regardless of radiation dose. As the diagnostic performance for the 30 cases was reasonably high for the attending radiologists and senior residents (with pooled AUC of 0.92–0.94), the investigators suggested that the clinical implementation of the 2 mSv CT would be feasible in many hospitals without further education, assuming that qualified site radiologists carefully supervise the practice.

### 3.4. Dose Calibration and Monitoring

**Figure 2:**
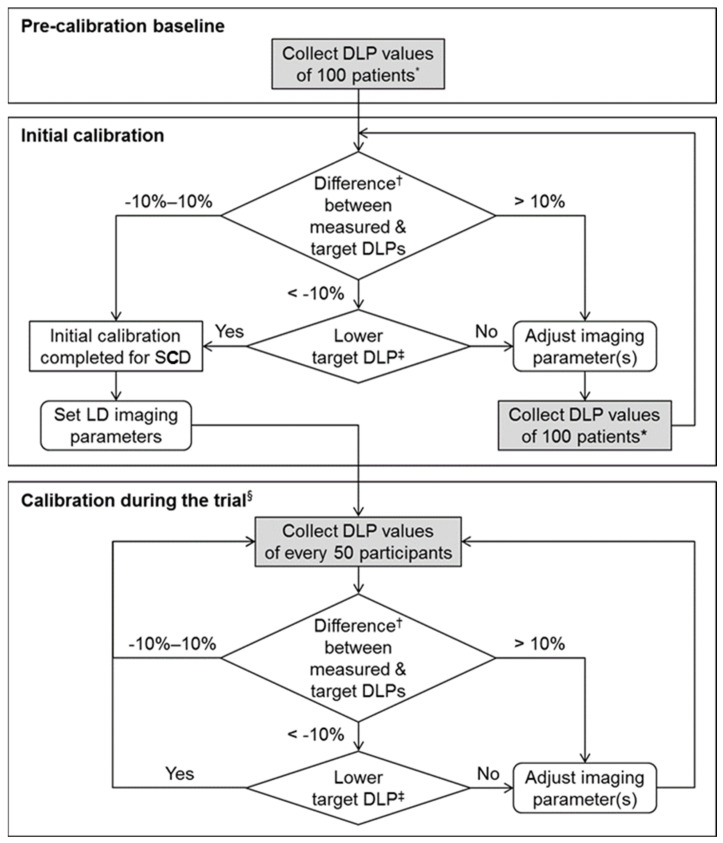
Radiation dose calibration procedures for each CT machine. * In regular abdomen CT examinations for various purposes in patients not enrolled in the trial. ^†^ Difference (%) = (measured median DLP—target DLP)/target DLP × 100. ^‡^ At the discretion of the lead radiologist. ^§^ For each of conventional-dose and low-dose groups. DLP = dose-length product, LD = low dose, SCD = site conventional dose.

While there have been a few studies on the principle of low-dose scan techniques [43], they have rarely addressed the specific step-by-step procedures on how to adjust the scanning parameters to reach and maintain the desired dose. Because different CT machines use different mechanisms of dose adjustment and automatic exposure control, there cannot be a single correct guideline. Here, we introduce the dose calibration and monitoring procedures that we developed as a part of the protocol [34] of the multi-center randomized clinical trial [4] (Figure 2).

Since we use automatic exposure control techniques, the actual radiation dose varies substantially with individual patient size. For each patient, the modulated radiation dose in terms of CTDI_vol_ (based on the use of 32 cm diameter reference phantom) and DLP is recorded as a text table in a Digital Imaging and Communications in Medicine image. If an additional scan is performed for any reason (e.g., rescan for nondiagnostic initial scan, machine failure, or extravasation), then the DLP for each helical scan is recorded. For an average-size patient, we initially set the target DLP as 130 mGy·cm for each scan, which corresponds to the effective dose of 2 mSv with a conversion factor of 0.015 mSv·mGy^–1^·cm^–1^ [44]. As we discussed earlier, we chose this initial dose level based on the previous studies that directly compared LDCT and CDCT. In each CT scanner, scan parameters such as reference tube-current-time products (or noise level) and tube potential are adjusted aiming at the target DLP value [45], and the parameter set is saved as an automated scan program. In general, the DLP value is roughly proportional to reference tube-current-time products but is nonlinear to the change of tube potential.

The target dose level can gradually decrease to some extent over time with advances in CT technology and radiologists’ adaptation to noisy images. Therefore, we have a unidirectional standpoint in resetting the target radiation dose: being flexible toward a lower dose while being strict against a higher dose (Figure 2). For each CT machine, we draw a box-and-whisker plot of the DLP distribution in a sizable group (e.g., 50) of consecutive patients to ensure appropriate calibration. We calculate the median DLP while excluding outliers caused by inappropriate scan techniques or technical failures. If the median DLP value is less than 90% or greater than 110% of the predefined target DLP (i.e., out of the error range of ±10% from the target DLP), we readjust the scan parameters (e.g., reference tube-current-time product or noise level) as appropriate. The calibration and monitoring processes are then iterated for every 50 patients for each CT machine.

## 4. Conclusions

Applying research achievements to clinical practice often requires more effort than that for the research itself. For successful implementation of LDCT practice, it is helpful to organize a team of radiologists, referring physicians or surgeons, and CT technologists, each of whom can champion the change toward LDCT and educate colleagues in their field.

## Data Availability

Not applicable.

## Supplementary Materials

The following supporting information can be downloaded at: https://www.mdpi.com/article/10.3390/diagnostics12071585/s1, Video S1: 36-year-old man with acute appendicitis who underwent contrast-enhanced CT scan. Stack mode display of 5-mm-thick transverse sections with a 4-mm increment (left). Sliding-slab averaging mode display (5-mm slab thickness) of the source dataset (2-mm thick at 1-mm increments) in the transverse plane (right). Video S2: 36-year-old man with acute appendicitis who underwent contrast-enhanced CT scan. Stack mode display of 5-mm-thick coronal sections with a 4-mm increment (left). Sliding-slab averaging mode display (5-mm slab thickness) of the source dataset (2-mm thick at 1-mm increments) in the coronal plane (right).

## References

[1] Lee K.H., Lee S., Park J.H., Lee S.S., Kim H.Y., Lee W.J., Cha E.S., Kim K.P., Lee W., Lee J.Y. (2021). Risk of hematologic malignancies from abdominopelvic CT radiation in patients who underwent appendectomy: A nationwide population-based cohort study. JAMA Surg..

[2] Park J.H., Salminen P., Tannaphai P., Lee K.H. (2022). Low-dose abdominal CT for evaluating suspected appendicitis in adolescents and young adults: Review of evidence. Korean J. Radiol..

[3] Kim K., Kim Y.H., Kim S.Y., Kim S., Lee Y.J., Kim K.P., Lee H.S., Ahn S., Kim T., Hwang S.S. (2012). Low-dose abdominal CT for evaluating suspected appendicitis. N. Engl. J. Med..

[4] LOCAT Group (2017). Low-dose CT for the diagnosis of appendicitis in adolescents and young adults (LOCAT): A pragmatic, multicentre, randomised controlled non-inferiority trial. Lancet Gastroenterol. Hepatol..

[5] Kim H.J., Lee K.H., Kim M.J., Park S.B., Ko Y. (2020). Using 2-mSv appendiceal CT in usual practice for adolescents and young adults: Willingness survey of 579 radiologists, emergency physicians, and surgeons from 20 hospitals. Korean J. Radiol..

[6] Garcia E.M., Camacho M.A., Karolyi D.R., Kim D.H., Cash B.D., Chang K.J., Feig B.W., Fowler K.J., Kambadakone A.R., Lambert D.L. (2018). ACR Appropriateness Criteria^®^ right lower quadrant pain-suspected appendicitis. J. Am. Coll. Radiol. JACR.

[7] Keyzer C., Tack D., de Maertelaer V., Bohy P., Gevenois P.A., Van Gansbeke D. (2004). Acute appendicitis: Comparison of low-dose and standard-dose unenhanced multi-detector row CT. Radiology.

[8] Keyzer C., Cullus P., Tack D., De Maertelaer V., Bohy P., Gevenois P.A. (2009). MDCT for suspected acute appendicitis in adults: Impact of oral and IV contrast media at standard-dose and simulated low-dose techniques. AJR Am. J. Roentgenol..

[9] Platon A., Jlassi H., Rutschmann O.T., Becker C.D., Verdun F.R., Gervaz P., Poletti P.A. (2009). Evaluation of a low-dose CT protocol with oral contrast for assessment of acute appendicitis. Eur. Radiol..

[10] Seo H., Lee K.H., Kim H.J., Kim K., Kang S.B., Kim S.Y., Kim Y.H. (2009). Diagnosis of acute appendicitis with sliding slab ray-sum interpretation of low-dose unenhanced CT and standard-dose intravenous contrast-enhanced CT scans. AJR Am. J. Roentgenol..

[11] Park J.H., LOCAT Group (2014). Diagnostic imaging utilization in cases of acute appendicitis: Multi-center experience. J. Korean Med. Sci..

[12] Pooler B.D., Lawrence E.M., Pickhardt P.J. (2012). Alternative diagnoses to suspected appendicitis at CT. Radiology.

[13] Dym R.J., Duncan D.R., Spektor M., Cohen H.W., Scheinfeld M.H. (2014). Renal stones on portal venous phase contrast-enhanced CT: Does intravenous contrast interfere with detection?. Abdom. Imaging.

[14] Preminger G.M., Tiselius H.G., Assimos D.G., Alken P., Buck C., Gallucci M., Knoll T., Lingeman J.E., Nakada S.Y., Pearle M.S. (2007). 2007 guideline for the management of ureteral calculi. J. Urol..

[15] Drake F.T., Alfonso R., Bhargava P., Cuevas C., Dighe M.K., Florence M.G., Johnson M.G., Jurkovich G.J., Steele S.R., Symons R.G. (2014). Enteral contrast in the computed tomography diagnosis of appendicitis: Comparative effectiveness in a prospective surgical cohort. Ann. Surg..

[16] Rao P.M., Rhea J.T., Novelline R.A., Mostafavi A.A., Lawrason J.N., McCabe C.J. (1997). Helical CT combined with contrast material administered only through the colon for imaging of suspected appendicitis. AJR Am. J. Roentgenol..

[17] Fefferman N.R., Roche K.J., Pinkney L.P., Ambrosino M.M., Genieser N.B. (2001). Suspected appendicitis in children: Focused CT technique for evaluation. Radiology.

[18] Brassart N., Winant C., Tack D., Gevenois P.A., De Maertelaer V., Keyzer C. (2013). Optimised z-axis coverage at multidetector-row CT in adults suspected of acute appendicitis. Br. J. Radiol..

[19] Kamel I.R., Goldberg S.N., Keogan M.T., Rosen M.P., Raptopoulos V. (2000). Right lower quadrant pain and suspected appendicitis: Nonfocused appendiceal CT--review of 100 cases. Radiology.

[20] Jacobs J.E., Birnbaum B.A., Macari M., Megibow A.J., Israel G., Maki D.D., Aguiar A.M., Langlotz C.P. (2001). Acute appendicitis: Comparison of helical CT diagnosis focused technique with oral contrast material versus nonfocused technique with oral and intravenous contrast material. Radiology.

[21] Zanca F., Demeter M., Oyen R., Bosmans H. (2012). Excess radiation and organ dose in chest and abdominal CT due to CT acquisition beyond expected anatomical boundaries. Eur. Radiol..

[22] Lee C.H., Goo J.M., Ye H.J., Ye S.J., Park C.M., Chun E.J., Im J.G. (2008). Radiation dose modulation techniques in the multidetector CT era: From basics to practice. Radiogr. A Rev. Publ. Radiol. Soc. N. Am. Inc..

[23] Coakley F.V., Gould R., Yeh B.M., Arenson R.L. (2011). CT radiation dose: What can you do right now in your practice?. AJR Am. J. Roentgenol..

[24] Lee K.H., Lee J.M., Moon S.K., Baek J.H., Park J.H., Flohr T.G., Kim K.W., Kim S.J., Han J.K., Choi B.I. (2012). Attenuation-based automatic tube voltage selection and tube current modulation for dose reduction at contrast-enhanced liver CT. Radiology.

[25] Willemink M.J., Leiner T., de Jong P.A., de Heer L.M., Nievelstein R.A., Schilham A.M., Budde R.P. (2013). Iterative reconstruction techniques for computed tomography part 2: Initial results in dose reduction and image quality. Eur. Radiol..

[26] Park J.H., Kim B., Kim M.S., Kim H.J., Ko Y., Ahn S., Karul M., Fletcher J.G., Lee K.H. (2016). Comparison of filtered back projection and iterative reconstruction in diagnosing appendicitis at 2-mSv CT. Abdom. Radiol..

[27] Park J.H., Jeon J.J., Lee S.S., Dhanantwari A.C., Sim J.Y., Kim H.Y., Lee K.H. (2018). Can we perform CT of the appendix with less than 1 mSv? A de-escalating dose-simulation study. Eur. Radiol..

[28] Johnson P.T., Horton K.M., Kawamoto S., Eng J., Bean M.J., Shan S.J., Fishman E.K. (2009). MDCT for suspected appendicitis: Effect of reconstruction section thickness on diagnostic accuracy, rate of appendiceal visualization, and reader confidence using axial images. AJR Am. J. Roentgenol..

[29] Sprawls P. (1992). AAPM tutorial. CT image detail and noise. Radiogr. A Rev. Publ. Radiol. Soc. N. Am. Inc..

[30] Paulson E.K., Harris J.P., Jaffe T.A., Haugan P.A., Nelson R.C. (2005). Acute appendicitis: Added diagnostic value of coronal reformations from isotropic voxels at multi-detector row CT. Radiology.

[31] Lee K.H., Kim Y.H., Hahn S., Lee K.W., Kim T.J., Kang S.B., Shin J.H. (2006). Computed tomography diagnosis of acute appendicitis: Advantages of reviewing thin-section datasets using sliding slab average intensity projection technique. Investig. Radiol..

[32] Lee Y.J., Kim B., Ko Y., Cho K.E., Hong S.S., Kim D.H., Song H., Lee K.H. (2015). 2-mSv CT in adolescents and young adults with suspected appendicitis: Advantages of additional review of thin sections using multiplanar sliding-slab averaging technique. AJR Am. J. Roentgenol..

[33] Weisenthal K., Karthik P., Shaw M., Sengupta D., Bhargavan-Chatfield M., Burleson J., Mustafa A., Kalra M., Moore C. (2018). Evaluation of kidney stones with reduced-radiation dose CT: Progress from 2011–2012 to 2015–2016—Not there yet. Radiology.

[34] Ahn S., LOCAT Group (2014). LOCAT (low-dose computed tomography for appendicitis trial) comparing clinical outcomes following low- vs standard-dose computed tomography as the first-line imaging test in adolescents and young adults with suspected acute appendicitis: Study protocol for a randomized controlled trial. Trials.

[35] Aly N.E., McAteer D., Aly E.H. (2016). Low vs. standard dose computed tomography in suspected acute appendicitis: Is it time for a change?. Int. J. Surg..

[36] Yun S.J., Ryu C.W., Choi N.Y., Kim H.C., Oh J.Y., Yang D.M. (2017). Comparison of low- and standard-dose CT for the diagnosis of acute appendicitis: A meta-analysis. AJR Am. J. Roentgenol..

[37] Yoon H.M., Suh C.H., Cho Y.A., Kim J.R., Lee J.S., Jung A.Y., Kim J.H., Lee J.Y., Kim S.Y. (2018). The diagnostic performance of reduced-dose CT for suspected appendicitis in paediatric and adult patients: A systematic review and diagnostic meta-analysis. Eur. Radiol..

[38] Brown T.W., McCarthy M.L., Kelen G.D., Levy F. (2010). An epidemiologic study of closed emergency department malpractice claims in a national database of physician malpractice insurers. Acad. Emerg. Med. Off. J. Soc. Acad. Emerg. Med..

[39] Howell J.M., Eddy O.L., Lukens T.W., Thiessen M.E., Weingart S.D., Decker W.W. (2010). Clinical policy: Critical issues in the evaluation and management of emergency department patients with suspected appendicitis. Ann. Emerg. Med..

[40] Mayo-Smith W.W., Hara A.K., Mahesh M., Sahani D.V., Pavlicek W. (2014). How I do it: Managing radiation dose in CT. Radiology.

[41] Kim S.Y., Lee K.H., Kim K., Kim T.Y., Lee H.S., Hwang S.S., Song K.J., Kang H.S., Kim Y.H., Rhee J.E. (2011). Acute appendicitis in young adults: Low- versus standard-radiation-dose contrast-enhanced abdominal CT for diagnosis. Radiology.

[42] Yang H.K., Ko Y., Lee M.H., Woo H., Ahn S., Kim B., Pickhardt P.J., Kim M.S., Park S.B., Lee K.H. (2015). Initial performance of radiologists and radiology residents in interpreting low-dose (2-mSv) appendiceal CT. AJR Am. J. Roentgenol..

[43] Parakh A., Kortesniemi M., Schindera S.T. (2016). CT radiation dose management: A comprehensive optimization process for improving patient safety. Radiology.

[44] Deak P.D., Smal Y., Kalender W.A. (2010). Multisection CT protocols: Sex- and age-specific conversion factors used to determine effective dose from dose-length product. Radiology.

[45] Huda W., Mettler F.A. (2011). Volume CT dose index and dose-length product displayed during CT: What good are they?. Radiology.

