# Supplementary material for: Low-Dose Abdominal CT for Evaluating Suspected Appendicitis: Recommendations for CT Imaging Techniques and Practical Issues
Source: Diagnostics (Basel). 2022 Jun 29;12(7):1585. doi: 10.3390/diagnostics12071585 (PMC9320604; doi:10.3390/diagnostics12071585)
Supplement: Supplementary file 1 [file diagnostics-12-01585-s001.zip › Supplementary Materials-1763977-220620-01.pdf]

Supplementary Materials

Video S1: 36-year-old man with acute appendicitis who underwent contrast-enhanced CT scan. Stack mode display of 5-mm-thick transverse sections with a 4-mm increment (left). Sliding-slab averaging mode display (5-mm slab thickness) of the source dataset (2-mm thick at 1-mm increments) in the transverse plane (right).

Video S2: 36-year-old man with acute appendicitis who underwent contrast-enhanced CT scan. Stack mode display of 5-mm-thick coronal sections with a 4-mm increment (left). Sliding-slab averaging mode display (5-mm slab thickness) of the source dataset (2-mm thick at 1-mm increments) in the coronal plane (right).
